# Nonlinear dynamics of a dispersive anisotropic Kuramoto–Sivashinsky equation in two space dimensions

**DOI:** 10.1098/rspa.2017.0687

**Published:** 2018-03-28

**Authors:** R. J. Tomlin, A. Kalogirou, D. T. Papageorgiou

**Affiliations:** 1 Department of Mathematics, Imperial College London, SW7 2AZ, London, UK; 2 School of Mathematics, University of East Anglia, NR4 7TJ, Norwich, UK

**Keywords:** Kuramoto–Sivashinsky equation, spatio-temporal chaos, active dissipative–dispersive nonlinear PDE

## Abstract

A Kuramoto–Sivashinsky equation in two space dimensions arising in thin film flows is considered on doubly periodic domains. In the absence of dispersive effects, this anisotropic equation admits chaotic solutions for sufficiently large length scales with fully two-dimensional profiles; the one-dimensional dynamics observed for thin domains are structurally unstable as the transverse length increases. We find that, independent of the domain size, the characteristic length scale of the profiles in the streamwise direction is about 10 space units, with that in the transverse direction being approximately three times larger. Numerical computations in the chaotic regime provide an estimate for the radius of the absorbing ball in $L^{2}$ in terms of the length scales, from which we conclude that the system possesses a finite energy density. We show the property of equipartition of energy among the low Fourier modes, and report the disappearance of the inertial range when solution profiles are two-dimensional. Consideration of the high-frequency modes allows us to compute an estimate for the analytic extensibility of solutions in $C^{2}$. We also examine the addition of a physically derived third-order dispersion to the problem; this has a destabilizing effect, in the sense of reducing analyticity and increasing amplitude of solutions. However, sufficiently large dispersion may regularize the spatio-temporal chaos to travelling waves. We focus on dispersion where chaotic dynamics persist, and study its effect on the interfacial structures, absorbing ball and properties of the power spectrum.

## 1. Introduction

The one-dimensional (1D) Kuramoto–Sivashinsky equation (KSE) is

1.1 $$u_{t}+uu_{x}+u_{xx}+u_{xxxx}=0,$$

which we consider equipped with periodic boundary conditions on the interval [0,*L*], and an *L*-periodic initial condition *u*(*x*,0)=*u*_0_(*x*). Owing to the conservative nature of (1.1) and the presence of a Galilean invariance, attention may be restricted to zero-mean solutions. This equation, and variants in higher space dimensions, arise in the study of spatio-temporal organization in reaction–diffusion systems [1], the propagation of flame fronts [2–4] and thin film flows down a vertical plane [5]. Variants also arise in two-phase flows [6–8]. Moreover, it is found to emerge in numerous applications in physics, including plasma physics [9], ion sputtering [10] and chemical physics for the propagation of concentration waves [11,12]. A wide range of dynamical behaviours are observed depending on the length *L* of the periodic domain. Increasing *L* above 2*π* (below 2*π* all solutions converge uniformly to zero), steady states, travelling waves and time-periodic bursts are observed, with the onset of chaos for large enough *L* [13]. The scaling of the system energy with the length parameter can be quantified by considering the *L*-dependent radius of the absorbing ball in the space $L_{\mathrm{per}}^{2}([0,L])$ for solutions to (1.1); this is a bound on the $L^{2}$-norm of the solutions in the large-time limit, i.e.

1.2 $$\underset{t\to \infty }{lim sup}{\left(\int_{0}^{L}u^{2}\;dx\right)}^{1/2}\leq CP(L),$$

for an appropriately chosen *L*-independent constant *C*, and some function *P*(*L*). An estimate of this form was first constructed using a Lyapunov function approach for odd solutions of (1.1), giving *P*(*L*)=*L*^5/2^ [14], a result which was later improved and generalized to non-parity solutions, implying the existence of a finite-dimensional global attractor [15]. After many intermediate developments [16–19], the most recent analytical improvement to this bound shows that (1.2) is satisfied for all solutions to (1.1) with *P*(*L*)=*L*^*q*^ for any *q*>5/6 [20,21]. Numerical work provides strong evidence that the optimal estimate for (1.2) is given by *P*(*L*)=*L*^1/2^ [22]; this was shown to be sharp for steady solutions of (1.1) using a dynamical systems approach by proving the stronger property of uniform boundedness of solutions independent of *L* [23] (this $L^{\infty }$ bound is also seen numerically for the general time-dependent case). It was also noted that the energy of the lower Fourier modes was equipartitioned, or spread equally [22,24,25], and decays exponentially for the higher Fourier modes due to strong dissipation on small scales. These regimes are separated by a peak in energy corresponding to the most active Fourier mode (this is near the most linearly unstable mode). The distribution of energy among the Fourier modes appears to be invariant to the system size *L* in the chaotic regime, suggesting an invariant energy distribution in the thermodynamic limit as L→∞. Furthermore, the decay of the fast high-frequency modes provides an optimal lower bound on the strip of analyticity of a solution about the real axis [26].

In this paper, we present numerical results for the spatially periodic initial value problem for a KSE in two space dimensions over rectangles *Q*=[0,*L*_1_]×[0,*L*_2_], given by

1.3 $$u_{t}+uu_{x}+u_{xx}+\delta \Delta u_{x}+\Delta^{2}u=0,$$

with periodic initial condition *u*(*x*,*y*,0)=*u*_0_(*x*,*y*) and dispersion parameter *δ*≥0. This was derived by Nepomnyashchy [27,28] with *δ*=0, and in general by Frenkel & Indireshkumar [29] and Topper & Kawahara [30] to describe the weakly nonlinear evolution of the interface of a thin film flow down a vertical plane (see [31] for a discussion of the derivations of this model for different fluid dynamical regimes). Without loss of generality, we can restrict our attention to zero-mean solutions because the spatial average of a solution to (1.3) is conserved and the equation is invariant under a Galilean transformation as in the 1D case. In the absence of dispersion, i.e. *δ*=0, equation (1.3) was studied analytically by Pinto [32,33] in the case of *L*_1_=*L*_2_=*L*. He proved global existence of solutions, the existence of a compact global attractor and analyticity of solutions. Using the Lyapunov function method, he obtained the estimate for the radius of the absorbing ball in $L_{\mathrm{per}}^{2}({\left[0,L\right]}^{2})$,

1.4 $$\underset{t\to \infty }{lim sup}∥u{∥}_{2}\leq CL^{12}\ln\;L,$$

where, in terms of general domain lengths *L*_1_ and *L*_2_, we define the $L^{2}$-norm by

1.5 $$∥u{∥}_{2}^{2}=\int_{0}^{L_{1}}\int_{0}^{L_{2}}u^{2}\;dx\;dy=L_{1}L_{2}\sum_{\underline{k}\in Z^{2}}|u_{\underline{k}}{|}^{2},$$

where $u_{\underline{k}}$ are the Fourier coefficients of *u*. The non-dispersive problem was also considered numerically by Akrivis *et al.* [34] on a square domain. They found that

1.6 $$\underset{t\to \infty }{lim sup}∥u{∥}_{2}\leq CL,$$

which is a significant improvement on the analytical result (1.4). In this paper, we generalize this result to periodic rectangular domains. Using numerical results from a large range of aspect ratios, we conjecture that the optimal bound for the radius of the absorbing ball of solutions to (1.3) for *δ*=0 in the space $L_{\mathrm{per}}^{2}(Q)$ is given by

1.7 $$\underset{t\to \infty }{lim sup}∥u{∥}_{2}\leq CL_{1}^{1/2}L_{2}^{1/2}.$$

In fact, we see the stronger result that the $L^{\infty }$-norm of solutions is bounded independent of *L*_1_ and *L*_2_. The result (1.7) implies that the solutions in the chaotic regime possess a finite energy density. We obtain a similar picture for the energy distribution of the Fourier modes as is found for the 1D KSE (1.1); a plateau of the energy for the low modes, rising to a peak and then decaying exponentially for the higher Fourier modes. The addition of the extra dimension in the dissipative fourth-order term of (1.3) produces an asymmetric energy distribution. By considering the decay of the Fourier spectra for large wavenumbers, we observe an increased spatial analyticity due to two-dimensionality of the solutions on domains that are not thin.

Next, we introduce dispersion to the problem, and consider how a small positive value of *δ* affects the dispersionless solution dynamics, energy distribution and the absorbing ball estimate (1.7). Dispersive effects are often included in the 1D KSE (1.1); Akrivis *et al.* [35] considered the addition of both third- and fifth-order dispersion by studying the Benney–Lin equation

1.8 $$u_{t}+uu_{x}+u_{xx}+\delta u_{xxx}+u_{xxxx}+\mu u_{xxxxx}=0.$$

They observed that increasing dispersion regularizes chaotic dynamics and supports travelling wave attractors. In the case of third-order dispersion alone with *μ*=0 (which is of interest here), formal asymptotics show that for δ→∞, solutions of (1.8) converge to scaled travelling wave solutions of the Korteweg–de Vries (KdV) equation—this convergence has also been proved rigorously in [36], and the stability of the resulting travelling waves was studied in [37,38]. For system lengths yielding chaotic attractors, a reverse period-doubling cascade was observed as *δ* is increased (see fig. 4.2 in [35]). This laminarizing effect of dispersion in the 1D problem was additionally investigated by Chang *et al.* [39], where the authors showed that increasing dispersion diminishes the family of steady and travelling wave solutions—only KdV pulses remain for large enough *δ*, with a large basin of attraction. Gotoda *et al.* [40] studied the route of the full dynamics as dispersion is strengthened; they additionally estimated the critical value of *δ*≈0.2 (which appears to be independent of system length) where high-dimensional chaos crosses to low-dimensional chaos.

In this paper, we are interested in weak dispersive effects which do not regularize the chaotic dynamics; we study the effect of the fixed values of *δ*=0.01, 0.1, and 1 on the dynamics of the two-dimensional (2D) KSE (1.3). We provide numerical evidence that, given a fixed value of *δ*, the $L^{2}$-norm satisfies the bound

1.9 $$\underset{t\to \infty }{lim sup}∥u{∥}_{2}\leq C(\delta )L_{1}^{1/2}L_{2}^{1/2}.$$

We also look at the equipartition and analyticity properties as for the non-dispersive case. We do not study the large dispersion limit (*δ*≫1) here, but briefly comment on known results. Travelling wave attractors of 2D solitary pulses are found, as observed by Toh *et al.* [41] and Indireshkumar & Frenkel [42], in analogy with the results in [35] for the 1D equation. Saprykin *et al.* [43,44] also studied this problem on an infinite domain, and provided a detailed analysis of the interaction between pulses. It can be shown with formal asymptotics (proved rigorously in [45]) that the solutions of (1.3) in this large dispersion limit converge to solutions of the Zakharov–Kuznetsov equation

1.10 $$U_{\tau }+UU_{x}+\Delta U_{x}=0,$$

where *U* and *τ* are rescalings of *u* and *t*, respectively. This is a higher-dimensional KdV equation yielding 2D solitons whose stability has been studied analytically in [46,47].

A related equation of interest is the multi-dimensional KSE,

1.11 $$v_{t}+\frac{1}{2}|\nabla v{|}^{2}+\Delta v+\Delta^{2}v=0,$$

also considered on periodic domains. In two spatial dimensions, this equation has been derived to describe the propagation of a planar flame front [4], and has been suggested (with the addition of stochastic noise) as an empirical model for the evolution of surfaces eroded by ion bombardment [10,48,49]. A number of authors [50–52,53] have considered (1.11) analytically, proving global existence of solutions on sufficiently thin domains for restricted classes of initial conditions. Kalogirou *et al.* [54] provided a comprehensive numerical study to complement this analytical work. They give an exhaustive picture of the dynamics present for varying domain dimensions.

The structure of the paper is as follows. In §2, we briefly discuss the numerical schemes and data analysis tools employed for our simulations. The computations of (1.3) with *δ*=0 follow in §3, and the dispersive case follows in §4. In §5, we discuss our results and future work.

## 2. Numerical methods and data analysis tools

Equation (1.3) is solved numerically using implicit–explicit backwards differentiation formulae (BDF) for the time discretization, and spectral methods in space. The BDF belong to the family of linearly implicit methods constructed and analysed by Akrivis & Crouzeix [55] for a class of nonlinear parabolic equations. It was shown by Akrivis *et al.* [34] that such numerical schemes are convergent, and also that they are efficient and unconditionally stable under various conditions on the linear and nonlinear terms of the problem. We do not go into further details of these schemes here, because their applicability for our problem has been checked in [34]. As we are considering (1.3) on rectangular periodic domains, the solution may be written in the form of a Fourier series

2.1 $$u=\sum_{\underline{k}\in Z^{2}}u_{\underline{k}}(t)\;e^{i\underline{\tilde{k}}\cdot \underline{x}},$$

where $u_{\underline{k}}$ are the Fourier coefficients of *u*, and $\underline{\tilde{k}}=({\tilde{k}}_{1},{\tilde{k}}_{2})$ denotes the wavenumber vector with components defined by

2.2 $${\tilde{k}}_{1}=\frac{2\pi k_{1}}{L_{1}},\;{\tilde{k}}_{2}=\frac{2\pi k_{2}}{L_{2}},$$

for $\underline{k}\in Z^{2}$. As *u* is real-valued, $u_{\underline{k}}$ is the complex conjugate of $u_{-\underline{k}}$. For numerical simulations, we truncate this Fourier series to |*k*_1_|≤*M* and |*k*_2_|≤*N*, corresponding to a discretization of the spatial domain *Q* into (2*M*+1)×(2*N*+1) equidistant points.

The linear dispersion relation for (1.3) is

**Figure 1.**
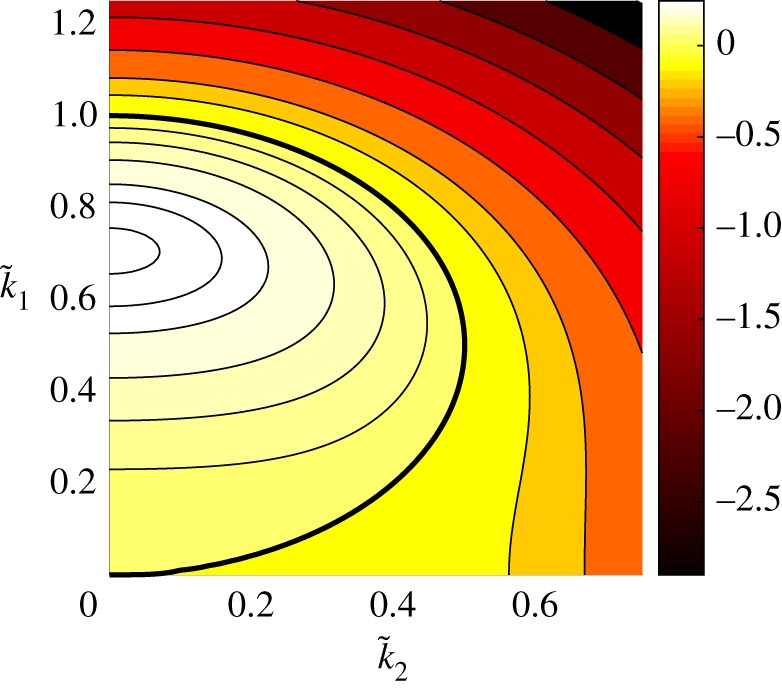
Contours of Re [s(k~_)] with the bold line corresponding to the zero contour, i.e. Re[s(k~_)]=0. The most linearly unstable mode is at $\tilde{\underline{k}}=(1/\sqrt{2},0)\approx (0.7071,0)$ where Re[s(k~_)]=1/4. For a given *L*_1_ and *L*_2_, the (*k*_1_,*k*_2_)-mode is linearly unstable/stable if the corresponding point $\left({\tilde{k}}_{1},{\tilde{k}}_{2}\right)$ lies inside/outside the zero contour. (Online version in colour.)

2.3 $$s(\underline{\tilde{k}})={\tilde{k}}_{1}^{2}+i\delta {\tilde{k}}_{1}({\tilde{k}}_{1}^{2}+{\tilde{k}}_{2}^{2})-{\left({\tilde{k}}_{1}^{2}+{\tilde{k}}_{2}^{2}\right)}^{2}={\tilde{k}}_{1}^{2}+i\delta {\tilde{k}}_{1}|\underline{\tilde{k}}{|}^{2}-|\underline{\tilde{k}}{|}^{4},$$

where the real part of $s(\underline{\tilde{k}})$ is the linear growth rate. The competition between the second-order destabilizing term and the fourth-order damping is clear from (2.3), yielding a region of linearly unstable wavenumbers for certain domain choices. Contours of the real part of *s*, as a function of ${\tilde{k}}_{1}$ and ${\tilde{k}}_{2}$, are shown in figure 1, where the zero contour is marked with a thicker line. For a fixed value of *L*_1_ and *L*_2_, the stability of the (*k*_1_,*k*_2_)-mode (where $\underline{k}\in Z^{2}$) is determined by the sign of the real part of $s(\underline{\tilde{k}})$, with linear instability for wavenumber vectors satisfying Re [s(k~_)]>0. It can be seen from figure 1 (and from the definition of *s*) that the purely transverse modes (*k*_1_=0) are always linearly stable. If *L*_1_≤2*π* (implying ${\tilde{k}}_{1}\geq 1$), we also see that no modes are linearly unstable because the real part of $s(\underline{\tilde{k}})$ is negative for all arguments; in this case, using an energy equation obtained by multiplying (1.3) by *u* and integrating over *Q*, it can be easily shown that the solution decays to zero exponentially. The purely imaginary component of $s(\underline{\tilde{k}})$ corresponds to the third-order dispersion term providing rotation of the Fourier coefficients in the complex plane. We may rewrite (1.3) as an infinite system of ODEs for the Fourier coefficients as

2.4 $$\frac{d}{dt}u_{\underline{k}}=-\frac{i{\tilde{k}}_{1}}{2}\sum_{\underline{m}\in Z^{2}}u_{\underline{k}-\underline{m}}u_{\underline{m}}+s(\underline{\tilde{k}})u_{\underline{k}}.$$

From this it can be seen that the purely transverse modes (${\tilde{k}}_{1}=0$) are unaffected by the nonlinear coupling and decay exponentially. However, the dynamics of the streamwise and mixed modes are slaved to the transverse modes through the nonlinear term, i.e. the transverse modes decouple partially.

We write the domain lengths *L*_1_,*L*_2_ in a canonical form, taking *L*_1_=*L* and *L*_2_=*L*^*α*^. We take α∈R in a range of values and vary *L* to present a view of the chaotic dynamics in the global attractor for many aspect ratios. For aspect ratios with *α*≤0, the domains are thin, as either *L*_1_≤1 or *L*_2_≤1. In the former case, we have trivial behaviour with solutions decaying to zero as mentioned before, and in the latter case only the purely streamwise modes may be linearly unstable, thus the dynamics of solutions are expected to be 1D (this is confirmed by numerical simulations). We use small-amplitude random initial conditions with unstable low wavenumber modes for our numerical simulations. For $\underline{x}=(x,y)\in Q$, we take

2.5 $$u_{0}(\underline{x})=\sum_{\begin{array}{c} |\underline{k}{|}_{\infty }=1\\ k_{1}\neq 0 \end{array}}^{20}a_{\underline{k}}\cos\;(\underline{\tilde{k}}\cdot \underline{x})+b_{\underline{k}}\sin\;(\underline{\tilde{k}}\cdot \underline{x}),$$

where the coefficients $a_{\underline{k}}$ and $b_{\underline{k}}$ are random numbers in the range [−0.05,0.05], generated separately for each pair (*L*,*α*). Owing to the existence of a global attractor [32], the large-time behaviour is independent of initial condition. Note that (2.5) does not contain contributions from the purely transverse modes (the summation excludes modes with *k*_1_=0). For large values of *L*_2_, the lower transverse modes have very small exponential decay rates and would affect the streamwise and mixed-mode dynamics at large times; taking transverse modes in the initial condition would only extend the transient phase of the dynamics.

We average desired quantities over one solution orbit in order to obtain an average of that quantity over the entire global attractor (orbits are assumed to be dense in the attractor). Instead of computing an estimate for

2.6 $$\underset{t\to \infty }{lim sup}∥u{∥}_{2}^{2},$$

we compute (as an equivalent) the time average of the energy defined by

2.7 $$E_{L,\alpha }=\lim_{T\to \infty }\frac{1}{T}\int_{0}^{T}∥u{∥}_{2}^{2}\;dt$$

and approximate it by

2.8 $${\bar{E}}_{L,\alpha }(T_{1},T_{2})=\frac{1}{T_{2}-T_{1}}\int_{T_{1}}^{T_{2}}∥u{∥}_{2}^{2}\;dt,$$

where 0≪*T*_1_≪*T*_2_ are two large times. We require *T*_1_ to be large enough that the solution has reached the global attractor, and *T*_2_ to be large enough that ${\bar{E}}_{L,\alpha }$ is a good enough approximation of the time average. For all numerical results, we chose *T*_1_=1×10^4^ and *T*_2_=2×10^4^, which proved to be suitable. To study the equipartition and analyticity properties, we consider the time-averaged power spectrum of solutions, given by

2.9 $$S(\underline{k})=L_{1}L_{2}\lim_{T\to \infty }\frac{1}{T}\int_{0}^{T}|u_{\underline{k}}{|}^{2}\;dt,$$

for each $\underline{k}\in Z^{2}$. Realistically, we approximate $S(\underline{k})$ by $\bar{S}(\underline{k};T_{1},T_{2})$ where we take a time average over [*T*_1_,*T*_2_] as done for ${\bar{E}}_{L,\alpha }$. We can visualize $\bar{S}(\underline{k})$ as a surface through discrete points, or we can condense the data by plotting the power spectrum against the magnitude of the wavenumber vector $|\underline{\tilde{k}}|={\left({\tilde{k}}_{1}^{2}+{\tilde{k}}_{2}^{2}\right)}^{1/2}$. Note that the energy *E*_*L*,*α*_ is related to $S(\underline{k})$ through

2.10 $$E_{L,\alpha }=\sum_{\underline{k}\in Z^{2}}S(\underline{k})$$

and we have the same relation between the approximate quantities ${\bar{E}}_{L,\alpha }$ and $\bar{S}(\underline{k})$.

## 3. Computations in the absence of dispersion

**Figure 2.**
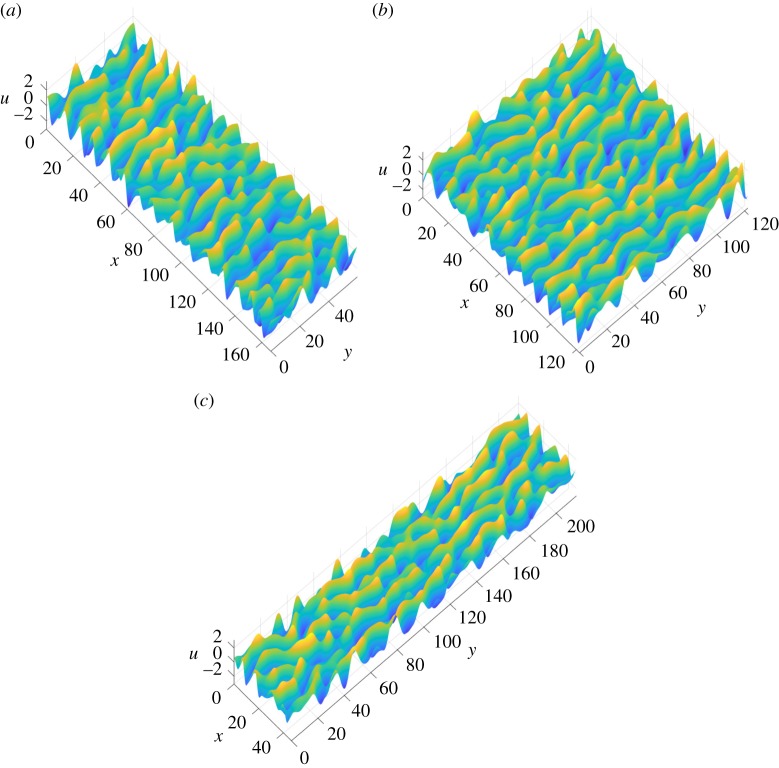
Profiles of numerical solutions to (1.3) with *δ*=0 in the chaotic regime at time *T*_2_=2×10^4^ are shown for a range of aspect ratios. The choice of domain dimensions are: (*a*) *L*=*L*_1_=166.81, *L*_2_=59.95, *α*=0.8, |*Q*|=10^4^, (*b*) *L*=*L*_1_=*L*_2_= 122.474, *α*=1, |*Q*|=1.5×10^4^ and (*c*) *L*=*L*_1_=46.415, *L*_2_=215.44, *α*=1.4, |*Q*|=10^4^. The structure of the profiles appears to be invariant of the length scales (as long as the domain is not thin), and the characteristic length of the cellular structures in the *y*-direction is comparatively larger than in the *x*-direction. (Online version in colour.)

We first proceed with a numerical study of (1.3) with *δ*=0 on large periodic domains. As noted earlier, for *α*≤0, we either obtain trivial dynamics or 1D solutions corresponding to solutions of the 1D KSE (1.1), so we focus on domains with *α*>0 (not thin). Figure 2 shows instantaneous interfacial profiles of solutions in the chaotic regime at time *T*_2_=2×10^4^. A variety of aspect ratios are used: in figure 2*a* the domain is longer in the streamwise direction and has *L*_1_=166.8, *L*_2_=59.9 (i.e. *α*=0.8); figure 2*b* shows a solution on a square domain with *L*_1_=*L*_2_=122.5; and the domain in figure 2*c* is longer in the spanwise direction with *L*_1_=46.4, *L*_2_=215.4 (here *α*=1.4). In all cases shown, activity in the mixed modes promotes fully 2D solutions. These profiles highlight distinct features of solutions to (1.3) on sufficiently large domains; the behaviour is dominated by the streamwise dynamics, with solutions varying weakly in *y*, but maintaining the characteristic cellular behaviour in the *x*-direction associated with solutions to the 1D equation (1.1)—a streamwise slice of the solution profile is very similar to the typical profiles observed in the chaotic regime for the 1D equation. Movie S1 in the electronic supplementary material presents the time evolution of solutions to (1.3) for these aspect ratios; the solution profiles at the final time are those shown in figure 2. For all profiles shown in figure 2, the characteristic length of the nonlinear cellular structures in the streamwise direction is about 10 units; this corresponds to the most active streamwise Fourier mode which has a wavenumber slightly smaller than the most linearly unstable streamwise mode, ${\tilde{k}}_{1}=1/\sqrt{2}$. This shift to larger scales induced by the nonlinearity was also noted for the 1D problem by Wittenberg & Holmes [22]. No transverse modes are active at large times because they are linearly damped and unaffected by the nonlinear term; however, structures form in the spanwise direction due to the mixed mode activity—these structures have a length of approximately 30 space units. From a vast number of numerical experiments it appears that the characteristic cellular structures present in the profiles are independent of the aspect ratio and length parameters in the 2D chaotic regime. This is already evidence of extensive dynamics that are analogous to the 1D problem.

### (a) Computational estimation of the radius of the absorbing ball

**Figure 3.**
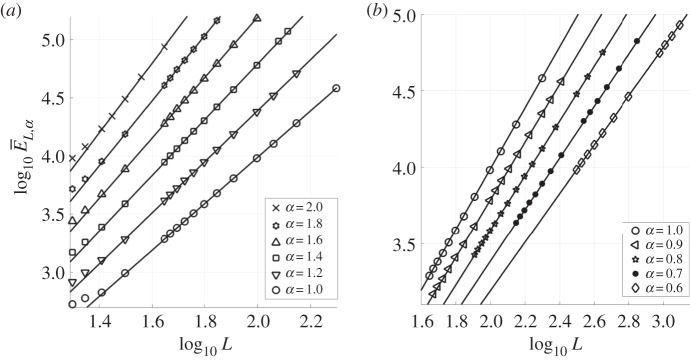
Plots of $\log_{10}\;{\bar{E}}_{L,\alpha }$ against $\log_{10}\;L$ for a range of *α*. Results for aspect ratios with 1≤*α*≤2, are shown in (*a*), and (*b*) considers 0.6≤*α*≤1. Best fit lines for each choice of *α* are also plotted—these are calculated using a least-squares approximation with the last few data points in each case.

In what follows, we present the results of extensive numerical experiments that were used to obtain an optimal numerical bound on the radius of the absorbing ball in $L_{\mathrm{per}}^{2}(Q)$; this generalizes the result (1.6) for square periodic domains (*α*=1) in [34]. To obtain the results that follow, *α* was fixed to take values in the interval 0.6≤*α*≤2, and *L* increased to cover a sufficiently large range of domains that support complex dynamics (recall that the rectangular domain has dimensions *L*×*L*^*α*^). For a given *α*, computations were carried out and the time-averaged quantity ${\bar{E}}_{L,\alpha }$ given by (2.8) was estimated for a range of values of *L*. We do not consider *α*≤0 because the dynamics are 1D as noted earlier. The variation of $\log_{10}\;{\bar{E}}_{L,\alpha }$ against $\log_{10}\;L$ is shown in figure 3. Figure 3*a* considers *α*≥1, i.e. domains that are longer in the spanwise direction, and figure 3*b* corresponds to *α*≤1, giving domains that are longer in the streamwise direction, with *α*=1 providing a reference between the two panels. We observe that a regime of direct proportionality between $\log_{10}\;{\bar{E}}_{L,\alpha }$ and $\log_{10}\;L$ emerges for sufficiently large length scales. It is apparent from our computations that the regime of linear proportionality arises when the shortest side of the periodic domain is greater than approximately 30.

A quantification of the linear behaviour apparent in figure 3 was carried out using a least-squares approximation to the slope of the different lines and their intercepts with the vertical axis. The slopes are found to be *α*+1 with an accuracy of 0.02 or less, and the vertical intercepts are all found to be zero, also with an accuracy of approximately 0.02. Hence, on sufficiently large domains with *α*>0, we observe that

3.1 $$\log_{10}\;E_{L,\alpha }\approx (1+\alpha )\log_{10}\;L,$$

which implies that *E*_*L*,*α*_≈*L*^1+*α*^. Surprisingly, the unit constant of proportionality in this expression for *E*_*L*,*α*_ is not found in the case of the 1D problem (1.1) where it is approximately 1.7. Recalling the definitions of *L*_1_ and *L*_2_, and with the established proportionality of *E*_*L*,*α*_ with the quantity $\underset{t\to \infty }{lim sup}∥u{∥}_{2}^{2}$, we obtain our optimal bound,

3.2 $$\underset{t\to \infty }{lim sup}∥u{∥}_{2}\leq CL^{(1+\alpha )/2}=CL_{1}^{1/2}L_{2}^{1/2},$$

where *C* is a constant which is independent of the length parameters. This result is also valid for *α*≤0, given the previous discussions on the dynamics in this regime. In fact, an even stronger result than (3.2) appears to hold; the numerical results provide evidence that the $L^{\infty }$-norm (the supremum of |*u*| over *Q* at a fixed time) of solutions in the chaotic regime is bounded above by a constant, in direct analogy with the numerical results for the 1D equation (1.1)—this can be seen in figure 2, where the solution amplitude appears to be independent of aspect ratio and length parameters. We find that the mean of the $L^{\infty }$-norm across the time series between times *T*_1_ and *T*_2_ is approximately 2.4, with the maximum value often being as large as 3.5; this result appears to be independent of *Q* as long as the domain is sufficiently large. This computational evidence that *u* is bounded by an *O*(1) constant (for example, 4 would suffice) over all choices of *Q* trivially implies (3.2).

### (b) Equipartition of energy and analyticity of solutions

In the previous subsection, we presented numerical evidence that predicts how the time-averaged energy of the system scales with the underlying lengths *L*_1_ and *L*_2_ for large domains supporting chaotic solutions. It is also particularly interesting to understand how this energy is spread among the Fourier modes. Recall that for the 1D KSE (1.1), it was observed that the energy was equipartitioned, i.e. spread equally among the lower Fourier modes (see [22] for example). The energy distribution rises to a peak for the most active mode (this is slightly less than the most linearly unstable mode) and then decays exponentially after an inertial range where the power spectrum behaves like $|\tilde{k}{|}^{-4}$. Interestingly, the energy distribution for the symmetric 2D KSE (1.11) also exhibits an inertial range, where the power spectrum behaves like $|\underline{\tilde{k}}{|}^{-6}$, and the exponent is also seen for the 1D form of (1.11)—this is natural given that (1.1) and the 1D form of (1.11) may be related through *u*=*v*_*x*_. This power law behaviour has been attributed to the balance of the destabilizing and dissipative linear terms for *O*(1) wavenumbers [24]. By the Paley–Wiener–Schwartz theorem (see [56] for example), the exponential decay of the high-frequency modes informs us of the spatial analyticity properties of solutions. For the 1D equation on an *L*-periodic domain, it is observed numerically that

3.3 $$|u_{k}|∼e^{-\beta (L)|\tilde{k}|},\;\mathrm{as}\;k\to \infty ,$$

where *β*(*L*) converges to approximately 3.5 as L→∞ [26]. This implies that we may extend the solution *u* analytically about the real axis into the complex plane in a strip with |*Im* *x*|<*β*(*L*). It is noted that *β*(*L*) converges to 3.5 from above (meaning that solutions lose spatial analyticity as *L* increases), and the limit value can thus be surmised to be the optimal lower bound of the width of the analytic extension.

**Figure 4.**
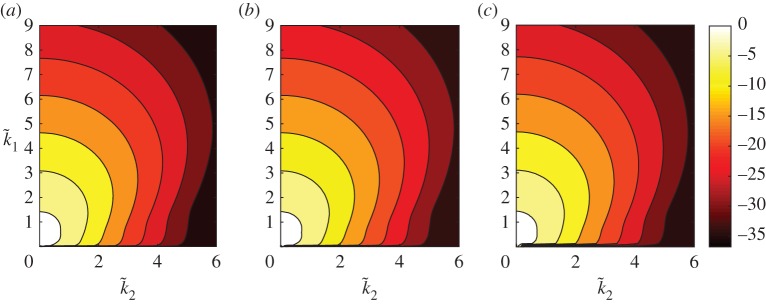
Contours of $\log_{10}\;\bar{S}(\underline{k})$ for a selection of aspect ratios with |*Q*|=10^4^. The domain dimensions are: (*a*) *L*=*L*_1_=166.81, *L*_2_=59.95, *α*=0.8, (*b*) *L*=*L*_1_=*L*_2_=100, *α*=1.0 and (*c*) *L*=*L*_1_=46.415, *L*_2_=215.44, *α*=1.4. Both (*a*) and (*c*) take the same dimensions for *Q* as (*a*) and (*c*) of figure 2, respectively. (Online version in colour.)

**Figure 5.**
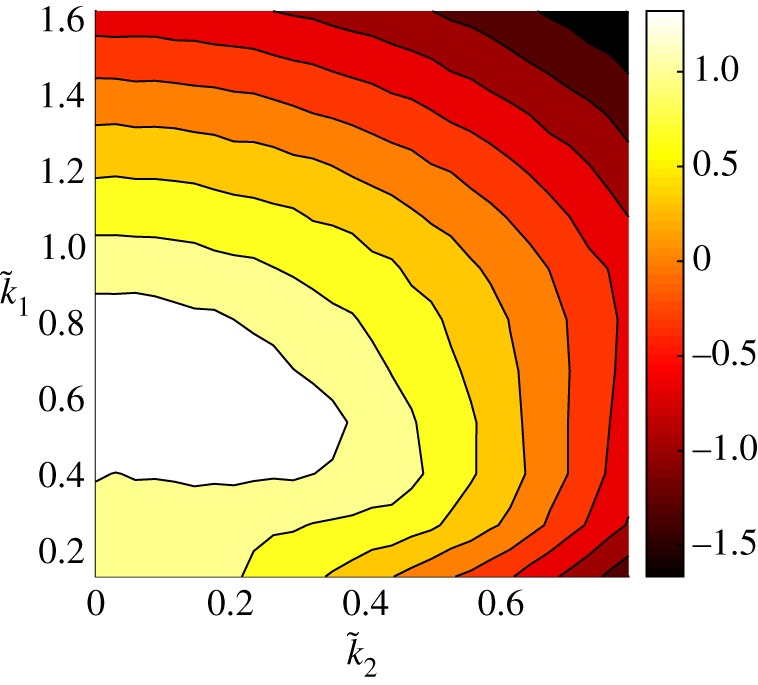
Contours of $\log_{10}\;\bar{S}(\underline{k})$ for *L*=*L*_1_=46.415, *L*_2_=215.44, *α*=1.4, |*Q*|=10^4^ (magnified view of figure 4*c*). For each ${\tilde{k}}_{1}$, the maximum value of the surface (corresponding to the most active mode) is found for ${\tilde{k}}_{2}=0$, with the streamwise modes with wavenumbers around 0.6 being the most active of all. A large tongue of active mixed modes with transverse wavenumbers up to approximately 0.3 is visible. (Online version in colour.)

For completeness and to check our numerical work, we have recovered the above results for (1.3) in the special limit that our periodic domain is thin in the transverse dimension, and we again concentrate on numerical results for aspect ratios with *α*>0. The key quantity is the time-averaged power spectrum $S(\underline{k})$ of the solutions given by (2.9) which is approximated by $\bar{S}(\underline{k})$ with an average over a finite time interval [*T*_1_,*T*_2_] as done for the energy in (2.8). Figure 4 depicts the spectrum $\bar{S}(\underline{k})$ for domains of different aspect ratios but equal areas |*Q*|=10^4^. The values of *α* used in figure 4 are 0.8, 1 and 1.4 for parts (*a*)–(*c*), respectively, and the corresponding values of *L* are 10^20/9^, 10^2^ and 10^5/3^ (recall that |*Q*|=*L*^1+*α*^). The respective streamwise–spanwise aspect ratios are 10^4/9^≈2.7826, 1 and 10^−2/3^≈0.2154. The figure shows logarithmic (base 10) contour plots for the three cases in the positive wavenumber quadrant corresponding to ${\tilde{k}}_{1},{\tilde{k}}_{2}\geq 0$. There are a number of noteworthy features of the results for the three representative domains selected: firstly, the contours are essentially equally spaced along rays from (0,0) as the exponent decreases to negative values, indicating that there is exponential decay of the power spectrum as ${\tilde{k}}_{1}$ and ${\tilde{k}}_{2}$ increase. The smallest exponential decay rate is observed in the streamwise (*k*_1_,0)-modes (the spacing between the contours is the largest in this direction). Secondly, the power spectrum remains *O*(1) as ${\tilde{k}}_{1}$ and ${\tilde{k}}_{2}$ become small, in analogue with the 1D equation (1.1). Lastly, and most noticeably from figure 4, the power spectra for all three cases appear to be almost identical, which suggests that, in the chaotic regime, the distribution of the energy among the Fourier modes is insensitive to the domain aspect ratio (assuming that the domain is not thin and mixed modes are active, as is the case for the choices of *Q* used to produce the figure). In figure 5, we zoom in on the low mode region of the power spectrum shown in figure 4*c*; the other cases provide similar plots. The tongue of most active modes (depicted by the white region in the figure) is consistent with the characteristic length scales of the cellular structures of profiles shown in figure 2. The most active streamwise mode with wavenumber 0.6 gives a length of approximately 2*π*/0.6≈10 space units. The longer length scale in the transverse direction is compatible with the observation that the tongue only extends to mixed modes with transverse wavenumbers around 0.3.

**Figure 6.**
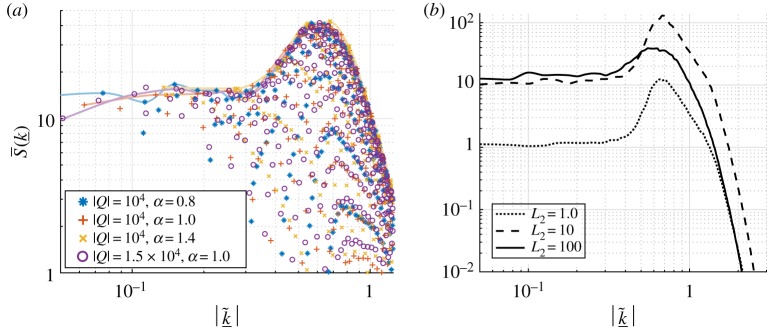
Equipartition of the energy. (*a*) The time-averaged power spectra $\bar{S}(\underline{k})$ of four sets of solution data plotted against the size of the wavenumber vector $|\underline{\tilde{k}}|$ on log–log axes. For |*Q*|=10^4^ we have three sets of data for different aspect ratios: *α*=0.8,1 and 1.4. For |*Q*|=1.5×10^4^ we have one set of data for *α*=1. The data points corresponding to the streamwise (*k*_1_,0)-modes are interpolated with cubic splines. All data points are from the quadrant corresponding to ${\tilde{k}}_{1}$, ${\tilde{k}}_{2}\geq 0$; the other quadrants give similar plots. (*b*) Comparison of the cubic spline interpolations of the streamwise mode spectrum for *L*_1_=250 and for the three choices of *L*_2_=1,10,100. (Online version in colour.)

Figure 6 provides a better description of the behaviour of the low modes, plotting the power spectrum against the size of the scaled wavenumber vector, $|\underline{\tilde{k}}|$. Figure 6*a* compares three different aspect ratios, with two sets of data for the square domain case—all simulations exhibit fully 2D chaotic dynamics. The purely streamwise modes are interpolated with a cubic spline which appears to bound the data points; these modes carry the most energy, which is unsurprising given the anisotropy of figure 4, and the fact that they are the most linearly unstable modes for a given value of $|\underline{\tilde{k}}|$. The equipartition of the energy is recovered for the streamwise modes—the interpolant plateaus for $|{\tilde{k}}_{1}|≲10^{-0.5}$. Furthermore, we see a peak in energy corresponding to the most active Fourier mode (as in the 1D case, this is slightly less than the most linearly unstable mode), and then the energy decays exponentially for large $|\underline{\tilde{k}}|$. Interestingly, the inertial range which is discernible for the 1D KSE is not seen in figure 6*a*; we propose that the disappearance of the inertial range is due to the mixed mode activity when the transverse length is sufficiently large. This is investigated in figure 6*b*, where for a fixed value of *L*_1_=250, we observe how increasing *L*_2_ affects the interpolant through the streamwise mode data points. For *L*_2_=1, 10, the mixed modes are not active in the solutions and the resulting dynamics are 1D—the solutions are just elongations of solutions to the 1D KSE (1.1) in the transverse direction. The dotted line in figure 6*b* corresponding to *L*_2_=1 matches the curve in [22] (for *L*_2_=1, the definition of the power spectrum (2.9) reduces to the definition for the 1D case), and the curve for *L*_2_=10 is simply a factor of 10 greater. Increasing *L*_2_ further, the spectrum begins to widen with increased activity in the mixed modes, and the streamwise component of the power spectrum tends towards the solid line shown in figure 6*b* for *L*_2_=100. For this choice of *L*_2_, the mixed modes are fully active in solutions, although we omit the data points lying on and below the interpolant in figure 6*b* because they are shown in figure 6*a*. Note also that no mixed modes are linearly unstable until approximately *L*_2_=40, although activity is seen for much smaller *L*_2_ due to the energy transfer through the nonlinear term; equivalently, the mixed modes are linearly unstable about 1D chaotic solutions for much smaller *L*_2_—this can be observed from a crude truncation of the set of ODEs for the Fourier modes (2.4). The inertial range (the linear behaviour for wavenumbers beyond the most active wavenumber) visible for *L*_2_=1, 10, is no longer discernible for *L*_2_=100, and the most active mode shifts even further towards the longer waves. This is consistent with the finding that the characteristic streamwise cell size of the profiles in figure 2 is larger than that found in simulations of the 1D equation, the respective values being 10 and 9 space units. Note that the equipartition observed in figure 6 is expected given its relation to the solution energy (2.10) which scales with |*Q*|—there is a constant energy density of solutions in the large domain limit.

The effect of the mixed mode activity can be seen more drastically in the analyticity of solutions. In [57], the authors give the generalization of the connection between the decay rate of the Fourier spectrum and the analyticity of solutions to the 2D case. Informally, the observation that

3.4 $$|u_{\underline{k}}|∼e^{-\beta (L_{1},L_{2})|\underline{\tilde{k}}|},\;\mathrm{as}\;|\underline{k}|\to \infty ,$$

implies that the function *u* may be extended holomorphically into $C^{2}$ in a ball of radius *β*(*L*_1_,*L*_2_). They also provide the analytical estimate for the problem (1.3) with *δ*=0 and *α*=1 that

3.5 $$\beta (L,L)\geq \tilde{C}L^{-1/5}{\left(\log\;L\right)}^{-2/3}.$$

This estimate depends on the length scales, as does the analytical estimate for the 1D equation [26]. As the streamwise modes yield the smallest exponential decay rate, an estimate for *β* may be computed numerically using a least-squares approximation from the slope of $-\log\;|u_{\left(k_{1},0\right)}|$ when plotted against $|{\tilde{k}}_{1}|$. Table 1 presents the results obtained for a range of domain dimensions. The optimal numerical lower bound on the strip of analyticity for solutions of the 1D KSE (1.1) is independent of *L* as mentioned earlier, and we find the same result in the 2D case, contrasting the analytical result (3.5). We recover the convergence of *β*(*L*_1_,*L*_2_) to approximately 3.5 for thin domains in the transverse dimension—the first two rows of table 1 take lengths *L*_1_ and *L*_2_ which result in no mixed mode activity, hence the dynamics are that of the 1D equation. We observe that increasing *L*_2_ so that mixed modes are active in the chaotic solutions actually improves the radius of analyticity, as observed in rows 3 to 7 of the table. For large domains with solutions exhibiting fully 2D spatio-temporal chaos, we are able to estimate that solutions can be extended holomorphically into $C^{2}$ in a ball of radius 3.8 approximately. Surprisingly, this decay rate appears for spectra just beyond the onset of fully 2D chaos and appears to remain relatively constant for all of our simulations with mixed mode activity. The present computations are very well resolved and yield values of *β* different from those obtained in [34] for the case of *α*=1, where the analyticity of solutions is found to be less than that observed for the 1D equation. Indeed, we find an increase in *β* from the 1D value, something which would be expected given the additional dissipation. Obviously, this does not improve the optimal lower bound on the analytic extensibility of solutions in the attractor, but it tells us that increasing two-dimensionality improves analyticity of the solutions (we assume that this is due to the activity of the mixed modes promoting energy in the dissipative range to move away from the purely streamwise modes).

**Table 1.** Estimates of decay rates of Fourier spectra, where *β*(*L*_1_,*L*_2_) is defined in (3.4).

*L*_1_	*L*_2_	*α*	|*Q*|	*β*
250	1	0	250	3.54
250	10	0.417	2.5×10^3^	3.55
46.415	215.44	1.4	10^4^	3.79
100	100	1	10^4^	3.80
166.81	59.95	0.8	10^4^	3.80
122.474	122.474	1	1.5×10^4^	3.81
250	100	0.834	2.5×10^4^	3.81

The 2D KSE (1.11) studied in [54] is symmetric, and the resulting power spectrum is thus a function of $|\underline{\tilde{k}}|$. For this problem, the radius of the ball of analytic extension in $C^{2}$ can be computed to be approximately 3.4. We note that the analyticity width for solutions to (1.3) is computed by considering the decay of the streamwise modes which are the most active, but due to the anisotropy of the spectrum, it is true that the solution may be extended further in different directions because the decay of the Fourier coefficients is asymmetric (for (1.3), the optimal analytic extension in $C^{2}$ is not a ball). This is in contrast with the problem (1.11), but we do not investigate this further here.

It is important to consider the possibility of a regime of dynamics beyond the length scales studied in this paper as discussed for the 1D problem (1.1) in [22]. It has been observed through extensive numerics and analysis that the large wavelength fluctuations of the 1D form of (1.11) ($v_{x}^{2}$ nonlinearity), can be described effectively by the Kardar–Parisi–Zhang (KPZ) equation [58], and correspondingly, the derivative form (1.1) can be described by a stochastically forced Burgers equation [59]. The inclusion of the 1D KSE with the $v_{x}^{2}$ nonlinearity in the so-called KPZ universality class is known as Yakhot’s conjecture [60], which correctly predicts that the roughness exponent is 1/2—the roughness exponent characterizes the scaling of the typical height fluctuations around the mean (of a saturated interface) with the length *L*, and is related to the $L^{2}$-norm of solutions. For the case of the $v_{x}^{2}$ nonlinearity, the interface width scales with *L*^1/2^ and the $L^{2}$-norm behaves like *L*. This scaling is observed for relatively small system sizes, although the other two critical exponents^1^ characterizing the KPZ universality class are not observed until *L* is much larger when full crossover to the KPZ scaling occurs. It is also worth noting that this asymptotic description is consistent with the observed energy spectrum. With this knowledge of the dynamics for the 1D problem, we conjecture that the energy behaviour (3.2) will not exhibit a crossover to a different scaling for even larger periodic domains. We do not attempt to compute the growth and dynamic exponents in this study, nor do we believe that the domain lengths used here are large enough to estimate these successfully; a number of studies have attempted to calculate these exponents for similar KS-type problems, but do so by resorting to very crude numerical discretizations in order to compute at large system sizes for a large number of time units. We are not certain that the form of the spectra observed for solutions in which mixed modes are active (figure 6) does not enter a different scaling regime which is computationally out of reach. In one of the less extreme cases used to compute the solution on a square domain with side *L*=100, there are 386 linearly unstable modes in total. This requires a numerical truncation with at least *M*=400, *N*=200 (approximately 320 000 Fourier modes) to obtain good accuracy (the spectrum is resolved to machine accuracy). Combining this with small time step requirements and large times of integration requires a large computing time.

## 4. Computations when dispersion is present

For the 1D KSE (1.1), it was observed in [35] that the strengthening of a physically derived third-order dispersion term can lead to a reverse period-doubling cascade. It is suggested that sufficiently large *δ* (i.e. a large amount of dispersion) can regularize chaotic dynamics for any system length *L*, as solutions are observed to be attracted to nonlinear travelling waves. Surprisingly, third-order dispersion acts as a destabilizing mechanism for this equation, competing with the stabilizing nonlinear term—it hinders the transfer of energy from low to high wavenumbers, and consequently analyticity of solutions reduces as *δ* is increased (see [61] for a discussion of this for the 1D case). Turning to the 2D problem, Toh *et al.* [41] and Indireshkumar & Frenkel [42] observed pulse solutions of (1.3) for large values of dispersion on large periodic domains—the usual streamwise cellular structures are found to be unstable and give way to the 2D pulses. An example of such a multi-pulse solution is given in Movie S2 in the electronic supplementary material, where the *O*(*δ*) pulses are seen to form an arrow head arrangement (the parameters taken are *L*_1_=*L*_2_=100 and *δ*=25). The arrow head of solitons is approximately time-periodic, with a period of about 10 time units; the pulses travel in the positive *x*-direction (streamwise) above a chaotic sea state of waves travelling upstream. Chaotic fluctuations of *O*(1) still exist in this case, but temporally periodic solutions are observed when *δ* is larger, where the *O*(1) component of solutions are time-periodic interactions at the bases of the pulses (see fig. 5 in [41]). We do not investigate the large *δ* limit here, nor questions concerning the regularization of chaotic dynamics. We are concerned with weak dispersive effects which do not fully regularize the chaotic behaviour, and observe how this affects the absorbing ball estimate and equipartition in the previous section.

**Figure 7.**
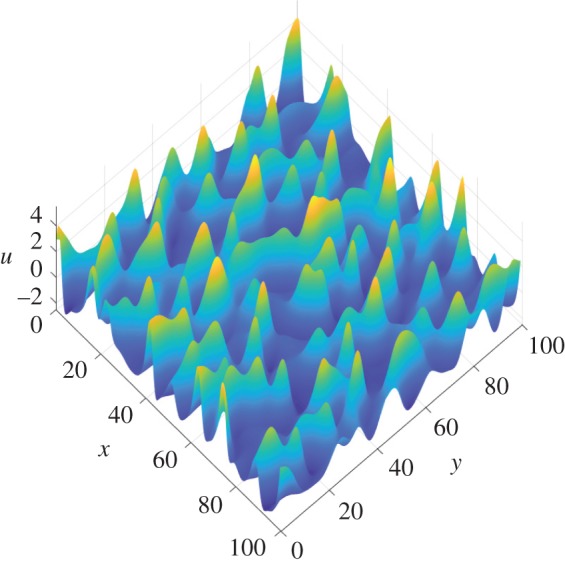
Profile of a numerical solution to (1.3) with *δ*=1 in the chaotic regime at time *T*_2_=2×10^4^. The dimensions of the periodic domain are *L*=*L*_1_=*L*_2_=100, *α*=1, |*Q*|=10^4^. Different aspect ratios produce similar profile structures; this was observed in figure 2 for the dispersionless case, so we do not plot other choices of *Q* here. (Online version in colour.)

The addition of dispersion qualitatively changes the profiles of solutions observed in the chaotic regime (see figure 2 for *δ*=0), yet they remain dominated by the streamwise dynamics as long as *δ* is not too large. The profile of a numerical solution in the chaotic attractor for *δ*=1 is shown in figure 7 for a square periodic domain with *L*=100. For *δ*=1, wavefronts are apparent, with higher peaks than the dispersionless case and flat trough regions in between. Streamwise slices of these profiles are similar to the solutions of 1D dispersive KS-type problems, for example the Benney–Lin equation (1.8)—the solutions observed are chaotic interactions of KdV pulses. These wavefronts cross and interact nonlinearly; this can be seen in Movie S3 in the electronic supplementary material, where the evolution of a solution to (1.3) with *δ*=1 is shown, and the profile at the final time is the same as that in figure 7. Our numerical simulations agree with the conjecture that chaotic dynamics may be regularized with sufficiently strong dispersion. We also note the path along which the regularization appears to occur as *δ* is increased: from the streamwise-dominated dynamics observed in the absence of dispersion, the cellular structures in the transverse direction begin to become more peaked in places forming wavefronts perpendicular to the streamwise direction. These fronts break up yielding pulse structures (for a square domain of side 100, this occurs around *δ*=5). Then, the arrangements of these 2D solitons are regularized fully to travelling waves for much larger *δ*.

**Figure 8.**
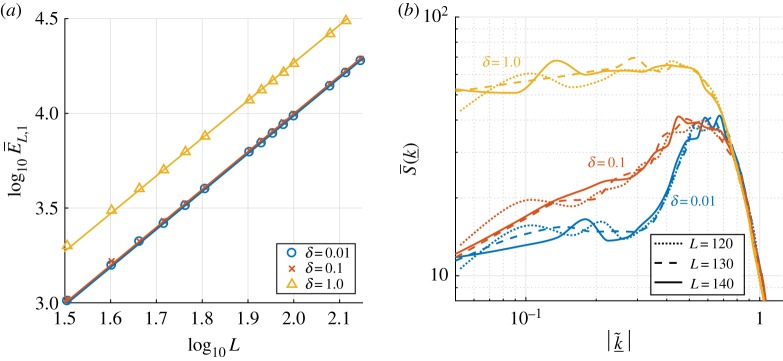
Energy behaviour for square domains (*α*=1) with *δ*=0.01,0.1,1. A plot of $\log_{10}\;{\bar{E}}_{L,1}$ against $\log_{10}\;L$ is shown in (*a*) (with best fit lines included), and (*b*) compares the interpolants through the streamwise data points for the power spectra on a log–log axis—for each *δ* we show three sets of data with *L*=120, 130, 140. (Online version in colour.)

In the dispersionless case, recall we observed that *E*_*L*,*α*_≈*L*^1+*α*^. To extend this result to the case of non-zero dispersion, we performed numerical simulations for *α*=0.8, 1 and 1.4, taking *δ*=0.01, 0.1 and 1. We obtained the same result as shown in figure 3, with a modification in the intercept of the straight lines with the vertical axis; this corresponds to the introduction of a constant $\tilde{C}(\delta )$ such that $E_{L,\alpha }\approx \tilde{C}(\delta )L^{1+\alpha }$. The case of *α*=1 with *δ*=0.01,0.1,1 is shown in figure 8*a*, and it is clear in this case, as in the other cases, that $\tilde{C}(\delta )$ increases monotonically in *δ* (the line for *δ*=0 is not included in figure 8*a* because it is unmistakeable from the *δ*=0.01 line at this scale). From our computations, we find roughly that $\tilde{C}(\delta )$ increases from 1 for *δ*∼*o*(1) to $\tilde{C}(1)\approx 2$. As before, this result yields the optimal numerical bound

4.1 $$\underset{t\to \infty }{lim sup}∥u{∥}_{2}\leq C(\delta )L_{1}^{1/2}L_{2}^{1/2}.$$

We also observe that the $L^{\infty }$-norm appears to be uniformly bounded as in the dispersionless case, and that this bound increases with *δ*; the chaotic profiles for larger values of dispersion have larger amplitude solutions, and the dynamics appears to consist of the creation, interaction and annihilation of many 2D pulses. The scaling of the $L^{\infty }$-norm with *δ* becomes linear in the large dispersion regime where the solution converges to travelling wave solutions of the ZKE (1.10), scaled by *δ*. Figure 8*b* shows how the increase of *δ* affects the energy distribution among the Fourier modes. The plot uses data from numerical simulations with *δ*=0.01, 0.1 and 1, for square domains of sides *L*=120, 130, 140, and shows the interpolants of the streamwise data points (these are found to be the most active modes as in the *δ*=0 case). Increasing *δ* results in a larger value of the small wavenumber asymptote and an increase in the energy in the low modes—this is consistent with the fact that *C*(*δ*) is an increasing function of its argument. The interpolant of the data points for *δ*=0.01 is almost identical to the dispersionless case shown in figure 6*a*, thus we do not plot the latter for comparison. For the moderate value of *δ*=0.1, the energy equipartition is skewed, as the active mode hump widens towards the low wavenumbers. The hump of active modes appears to cover the entire low wavenumber range for *δ*=1, and thus we recover the equipartition of energy among the low modes. For larger values of *δ* (for example, *δ*=25 as in Movie S2 in the electronic supplementary material), we recover peaks in the spectrum as observed for the 1D problem by Gotoda *et al.* [40]; however the mixed modes are much more active—further investigation of the dynamics of moderate to large *δ* is warranted.

In analogy with the 1D case, we see that the addition of dispersion decreases the radius of analyticity of solutions; for example, in the case of *δ*=1 and a domain which yields fully 2D solutions, it is observed that the Fourier coefficients decay as (3.4) with *β*≈3.5. As before, we find that the optimal numerical lower bound on the strip of analyticity occurs for thin domains (the smoothening of solutions due to two-dimensionality is independent of *δ*), and the corresponding 1D results are investigated in [35].

## 5. Conclusion

In this work, we have studied the dynamics of a physically derived dispersive KSE (1.3) in two spatial dimensions exhibiting extensive behaviour. Without dispersion, we observed that for sufficiently large domains, the system enters a regime of full spatio-temporal chaos, which is dominated by the streamwise dynamics (see Movie S1 in the electronic supplementary material). Furthermore, the system possesses a constant energy density since the $L^{2}$-norm of solutions scales with $|Q{|}^{1/2}=L_{1}^{1/2}L_{2}^{1/2}$. In keeping with this, we find that the energy distribution of the low modes converges to a constant surface as *L*_1_ and *L*_2_ become large (figure 4) and the $L^{\infty }$-norm of solutions is bounded independently of *Q*. These features are seen for the 1D KSE (1.1); however, the anisotropic 2D KSE (1.3) of interest in this paper does not present an inertial range in the simulations we have performed with mixed mode activity. In addition to this, we saw that the increase in two-dimensionality of solutions, through increasing the transverse length *L*_2_ until mixed modes become active, results in increased spatial analyticity. The optimal lower bound on the strip of analyticity is found when the domain is thin in the transverse direction, where the dynamics are governed by the 1D equation (1.1).

The addition of strong dispersion results in regularization of the spatio-temporal chaos, but moderate values of *δ* (dispersion parameter) change the nature of the chaotic dynamics, with interacting wavefronts that resemble KdV-type pulses emerging (see Movie S3 in the electronic supplementary material). The energy density is an increasing function of *δ*, and the constant $L^{\infty }$-norm bound on the solutions also increases with dispersion. As observed in 1D, dispersion has a destabilizing effect on the dynamics, as can be seen in a loss of spatial analyticity of solutions. Preliminary numerical runs indicate that (4.1) is valid in the large dispersion regime where the chaotic dynamics are regularized—the value of *δ* fixes the pulse height, and the number of pulses scales with the size of the periodic rectangular domain. Much larger values of dispersion require smaller time steps for good accuracy, and a comprehensive study of the moderate to large *δ* regime for very large domains is numerically challenging.

It appears that finite energy density, corresponding to systems exhibiting equipartition, is a hallmark of the dynamics of KS-type systems with a *uu*_*x*_ nonlinearity. This property has been shown for multi-dimensional equations even with the addition of dispersion and variation in the linear and nonlinear terms. A non-local KSE in 2D was derived by Tomlin *et al.* [62] for the problem of a gravity-driven thin liquid film under the action of a normal electric field. Preliminary results appear to indicate a finite energy density for this problem also. Current work by the authors is the investigation of the extent of the class of PDE with quadratic nonlinearities exhibiting a finite energy density (corresponding to a roughness exponent of 0) by considering non-local variants of (1.1). Correspondingly, there is the related problem of finding the extent of the KPZ universality class by considering equations with the $v_{x}^{2}$ nonlinearity.

## Footnotes

1 These are the growth and dynamic exponents which characterize the transient dynamics, i.e. before the solution orbit enters the chaotic attractor. These exponents are defined by how the surface roughness grows with time before saturation, and how the critical saturation time scales with the system lengths, respectively. Such exponents are not of current interest to us because we study large-time properties of solutions.

## Data accessibility

An executable file, datafile and Matlab script required to run and analyse numerical simulations of (1.3) are available at: https://github.com/RubenJTomlin/Anisotropic-dispersive-2D-Kuramoto-Sivashinsky-Equation.
